# Assessment of the Optic Nerve Sheath Diameter by Bedside Sonography and Its Correlation With the Glasgow Coma Score in the Outcome of Patients With Sepsis-Associated Encephalopathy

**DOI:** 10.7759/cureus.113361

**Published:** 2026-07-25

**Authors:** Kirtikar Ghorela, Raju Shakya, Mukul Garg, Shivani Kumawat

**Affiliations:** 1 Critical Care Medicine, Mahatma Gandhi Medical College and Hospital, Jaipur, IND; 2 Critical Care Medicine, Indraprastha Apollo Hospitals, New Delhi, IND; 3 Anaesthesiology, North Eastern Indira Gandhi Regional Institute of Health and Medical Sciences (NEIGRIHMS), Shillong, IND; 4 Pathology, Sanjay Gandhi Memorial Hospital, New Delhi, IND

**Keywords:** glasgow coma scale (gcs), intensive care unit, intracranial pressure (icp), optic nerve sheath diameter, sepsis-associated encephalopathy, ultrasonography (usg)

## Abstract

Background: Sepsis-associated encephalopathy (SAE) is an acute diffuse cerebral dysfunction occurring in patients with sepsis in the absence of direct central nervous system infection or another primary neurological disorder. Ultrasonographic measurement of the optic nerve sheath diameter (ONSD) has been proposed as a non-invasive surrogate marker of elevated intracranial pressure. This study evaluated the association of serial ONSD measurements with Glasgow Coma Scale (GCS) scores and clinical outcomes in patients with SAE.

Materials and methods: This prospective observational study was conducted in the Intensive Care Unit (ICU) of Indraprastha Apollo Hospitals, New Delhi, from May 2021 to October 2022. The study included 80 patients, aged ≥18 years, who were admitted to the ICU with SAE. ONSD and GCS scores were measured by bedside USG at the time of diagnosis of SAE (0 hrs), at 24 hours, 72 hours, on the fifth day, or when any clinical deterioration of the patient is evident. Outcomes measured were 28-day mortality, duration of ICU and hospital stays. Data thus collected were subjected to statistical analysis, and results were drawn.

Results: The mean age of the patients was 53.24 ± 13.20 years, and 42 patients (52.5%) were male. The mean Acute Physiology and Chronic Health Evaluation (APACHE) II and Sequential Organ Failure Assessment (SOFA) scores were 19.54 ± 3.33 and 9.32 ± 2.36, respectively. No statistically significant correlations were observed between ONSD and GCS scores at baseline, 24 hours, 72 hours, day 5, or the time of clinical deterioration. The baseline ONSD was not significantly correlated with the APACHE II score, SOFA score, ICU length of stay, or hospital length of stay. Twelve patients (15%) died within 28 days. The mean baseline ONSD was higher among non-survivors than among survivors (5.35 ± 0.19 versus 5.20 ± 0.23 mm; p=0.035). However, when the ONSD was dichotomized using a threshold of 5.5 mm, mortality was not significantly different between patients with elevated and normal ONSD values (30.0% versus 12.9%; p=0.16). No significant differences in ICU or hospital length of stay were identified according to the ONSD category.

Conclusion: Serial ONSD measurements were not significantly correlated with GCS scores, disease-severity scores, or length-of-stay outcomes in this cohort of patients with SAE. Although the mean baseline ONSD was higher among non-survivors, this finding was not reproduced when the ONSD was categorized using a 5.5-mm threshold and should therefore be considered exploratory. Larger studies using standardized ONSD protocols and adjusted longitudinal analyses are required.

## Introduction

Sepsis-associated encephalopathy (SAE) is an acute diffuse cerebral dysfunction that occurs in patients with sepsis without evidence of direct central nervous system infection or another primary neurological cause. Its clinical manifestations range from inattention, delirium, and behavioral changes to seizures, impaired consciousness, and coma. SAE is common among critically ill patients with sepsis and has been associated with increased mortality, prolonged hospitalization, persistent cognitive impairment, and reduced functional recovery [1-5]. The pathophysiology of SAE is multifactorial and may include neuroinflammation, endothelial dysfunction, disruption of the blood-brain barrier, impaired cerebral autoregulation, microcirculatory abnormalities, metabolic disturbances, and cerebral edema. In selected patients, these mechanisms may contribute to elevated intracranial pressure (ICP). Although invasive ICP monitoring remains the reference method for direct ICP measurement, it is not routinely indicated in patients with SAE because of its invasive nature and the associated risks of hemorrhage and infection [6].

The optic nerve is surrounded by a continuation of the intracranial subarachnoid space. Consequently, increases in cerebrospinal fluid pressure may result in distension of the retrobulbar optic nerve sheath. Ultrasonographic measurement of the optic nerve sheath diameter (ONSD) has therefore been investigated as a non-invasive surrogate for elevated ICP [7,8]. The technique is portable, repeatable, and feasible at the bedside; however, its diagnostic performance depends on the population studied, the measurement technique, operator experience, and the selected cutoff value. The ONSD should consequently be interpreted as an indirect marker rather than as a direct measurement of ICP.

Clinical severity scores, including the Sequential Organ Failure Assessment (SOFA) score [9], Acute Physiology and Chronic Health Evaluation (APACHE) II score [10], and Glasgow Coma Scale (GCS) [11], are commonly used to assess disease severity and prognosis in critically ill patients. Nevertheless, objective bedside markers of neurological dysfunction in SAE remain limited. Previous studies have reported conflicting findings regarding the relationship between ONSD, neurological status, and outcomes in patients with sepsis. Therefore, the present study aimed to evaluate the association of serial ultrasonographic ONSD measurements with GCS scores, APACHE II scores, SOFA scores, 28-day mortality, and the duration of intensive care unit and hospital stay in patients with SAE.

Thus, this study was conducted to investigate the correlation of the ONSD with the APACHE II score, SOFA score, GCS score, and outcomes (mortality and duration of ICU and hospital stay) in SAE patients admitted to our institute.

## Materials and methods

This prospective observational study included 80 adult patients admitted to the Intensive Care Unit (ICU) with SAE between May 2021 and October 2022. The bilateral ONSD was measured using bedside ultrasonography at the time of SAE diagnosis, 24 hours, 72 hours, day 5, and at the time of predefined clinical deterioration. GCS scores were recorded at the same time points by a clinician blinded to the ONSD measurements. The primary analysis evaluated the association between ONSD and GCS scores. Secondary outcomes included 28-day mortality and the duration of intensive care unit and hospital stay.

Inclusion criteria

Inclusion criteria were: (i) Age 18 yrs or more; (ii) patients admitted to the ICU with a diagnosis of sepsis based on positive culture reports; (iii) acute alteration in mental status temporally associated with sepsis; (iv) GCS score below 14 at the time of enrollment; (v) Absence of an alternative primary neurological, metabolic, toxic, or medication-related explanation for the alteration in consciousness.

Exclusion criteria

Exclusion criteria were: (i) Patients with orbital trauma; (ii) Patients with orbital mass; (iii) Patients with eye surgery; (iv) Patients with central nervous infections; (v) Patients with cerebrovascular accident/space occupying lesions; (vi) Presence of brain trauma or prior neurosurgery; (vii) Pregnant patients; (viii) Immunocompromised patients; (ix) Patients on chemotherapy or radiotherapy; (x) Patients on any psychiatric medications or sedatives.

SAE was operationally defined as an acute alteration in mental status occurring in temporal association with sepsis, with a GCS score below 14, after exclusion of direct central nervous system infection and other plausible causes of encephalopathy. Alternative causes considered included acute stroke, intracranial hemorrhage, traumatic brain injury, seizures or postictal states, hepatic or uremic encephalopathy, major electrolyte or glucose abnormalities, hypoxemia, hypercapnia, endocrine disorders, intoxication, medication effects, and sedative exposure. 

The study was done after getting clearance from the institutional ethical committee & after getting written informed consent from the relatives of patients. Demographic characteristics, comorbidities, source of infection, microbiological findings, organ-support requirements, APACHE II score, and SOFA score were recorded using a standardized case-report form. GCS scores were assessed at baseline, 24 hours, 72 hours, day 5, and at the time of clinical deterioration by a nurse or physician who was blinded to the ONSD results.

Bilateral ONSD measurements were performed by a trained intensivist experienced in point-of-care ultrasonography in patients diagnosed with SAE in ICU (0 hour) and later serial assessments at 24 hours, 72 hours, fifth day, or any clinical deterioration of the patient (drop in GCS by more than two). The GCS score was reported by an onsite nurse or another clinician who was blinded to the results of ONSD measurements, and it was also measured at 0 hour, 24 hours, 72 hours, fifth day, or at clinical deterioration of the patient to minimize interobserver bias.

ONSD measurement 

**Figure 1:**
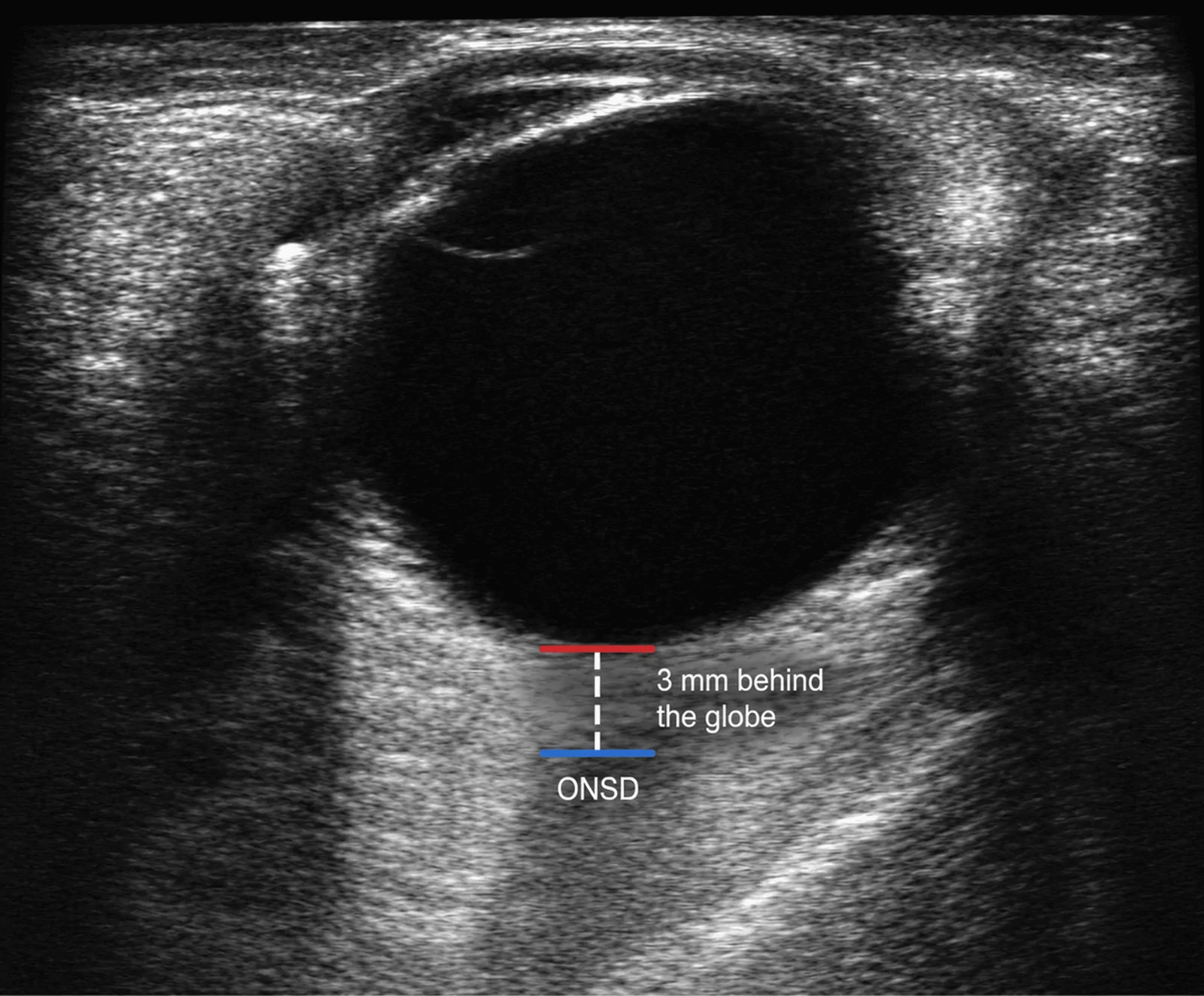
Optic nerve sheath diameter (ONSD) measured by a linear ultrasound probe Original image obtained using SonoSite M-Turbo (FUJIFILM Sonosite, Inc., Bothell, USA)

ONSD measurements were performed with the patient supine and head maintained in a neutral position. A Fujifilm SonoSite M-Turbo ultrasound system (FUJIFILM Sonosite, Inc., Bothell, USA) equipped with a [6-13]-MHz linear-array transducer was used. A small amount of sterile coupling gel was applied over the closed upper eyelid, and minimal pressure was exerted on the globe. The optic nerve was visualized as a hypoechoic structure extending posteriorly from the globe. ONSD was measured 3 mm posterior to the optic disc, from the outer edge of one dural sheath to the outer edge of the opposite sheath, using electronic calipers (Figure 1). Measurements were obtained in the transverse plane from both eyes. Each eye was measured three times, and the final ONSD value was calculated as an average of bilateral measurements. The operator was blinded to the GCS score, APACHE II score, SOFA score, and subsequent clinical outcome. The ultrasound output was maintained according to the as-low-as-reasonably-achievable principle and the safety recommendations of the British Medical Ultrasound Society [12].

A prespecified ONSD threshold of 5.5 mm was used for the categorical analyses, based on a previous study in patients with sepsis [13]. Values below 5.5 mm were classified as being below the threshold, whereas values of 5.5 mm or greater were classified as elevated according to the prespecified cutoff. Because ICP was not directly measured in the present study, this threshold was not considered diagnostic of intracranial hypertension (Figure 1).

Primary outcome

The primary outcome is the correlation of high and normal ONSD with 28-day mortality and GCS score.

Secondary outcome

The secondary outcome is association of high and normal ONSD with the length of hospital and ICU stay.

Sample size

The sample size was calculated using the correlation coefficient reported by Yang et al., who identified a correlation of −0.666 between ONSD and GCS score in patients with sepsis [13]. Using Fisher’s z transformation, a two-sided significance level of 0.05, 95% statistical power, and an expected absolute correlation coefficient of 0.666, the minimum estimated sample size was 24 patients. However, in view of the number of patients reporting to our hospital and also the flexibility of time, we collected data on 80 patients for this study. Further, such sample size led to an increase in power for the present study.

Statistical analysis

Statistical analysis was performed using IBM SPSS Statistics for Windows, Version 25 (Released 2017; IBM Corp., Armonk, New York, United States). The Mann-Whitney test, independent t-test, Pearson correlation coefficient, and Fisher’s exact test were used for analysis. A P-value <0.05 was considered statistically significant.

## Results

In this study, the mean age of study patients was 53.24±13.2 years, and the majority were male (52.5%). The mean APACHE II score and mean SOFA score were 19.54±3.33 and 9.32±2.36, respectively. The mean duration of ICU stay and hospital stay in study patients were 13.56±5.71 days and 18.23±7.3 days, respectively (Table 1).

**Table 1:** Characteristics of study patients Values are expressed as mean ± standard deviation or number (%).; ICU: Intensive care unit SOFA: Sequential Organ Failure Assessment; APACHE II: Acute Physiology and Chronic Health Evaluation II

Patient Characteristics	Mean ± SD (n=80)
Age (years)	53.24 ± 13.2
Male	42 (52.5%)
Female	38 (47.5%)
APACHE II score	19.54 ± 3.33
SOFA score	9.32 ± 2.36
ICU stay (days)	13.56 ± 5.71
Hospital stay (days)	18.23 ± 7.3

The mean ONSD (mm) and mean GCS score at 0 hour, 24 hours, 72 hours, and the fifth day are presented in Table 2. The mean ONSD did not vary at different time intervals, whereas the mean GCS score decreased over the period of time (Table 2).

**Table 2:** Descriptive statistics of mean ONSD (mm) and mean GCS score at different time intervals Values are expressed as mean ± standard deviation or number (%). GCS: Glasgow Coma Scale; ONSD: optic nerve sheath diameter

	Mean ± SD
Mean ONSD (mm)	
At 0 hour	5.22 ± 0.23
At 24 hours	5.22 ± 0.24
At 72 hours	5.22 ± 0.25
At 5th day	5.24 ± 0.25
Mean GCS Score	
At 0 hour	12.04 ± 0.83
At 24 hours	11.78 ± 1.26
At 72 hours	11.81 ± 1.4
At 5th day	11.41 ± 1.55
At clinical deterioration	10.29 ± 1.08
Day of deterioration	6.18 ± 2.89

The ONSD showed a non-significant mild negative correlation with the GCS at 0 hour and 24 hours, and no correlation at 72 hours and fifth day, whereas at the day of deterioration there was a non-significant mild positive correlation between the ONSD and GCS score (Table 3). 

**Table 3:** Correlation of the mean ONSD (mm) with GCS score at different time intervals GCS: Glasgow Coma Scale; ONSD: optic nerve sheath diameter

Time	R value	P-value
0 hour	-0.124	0.274
24 hours	-0.180	0.110
72 hours	0.032	0.779
5^th^ day	0.020	0.860
Day of deterioration	0.091	0.424

**Figure 2:**
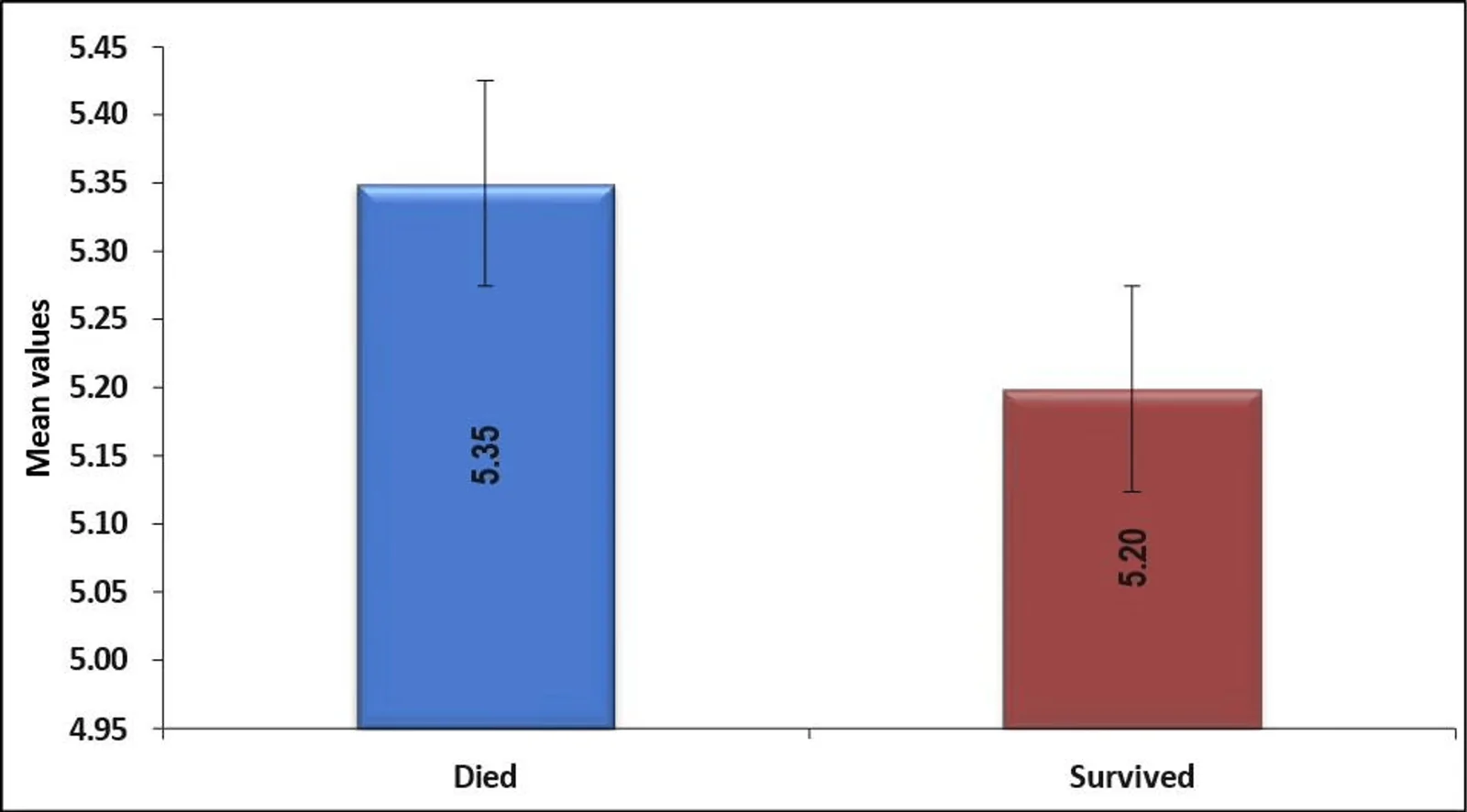
Association of the mean ONSD at 0 hour with 28-day mortality in study patients The graph was generated using SPSS software version 25.0 (IBM Corp., Armonk, NY, USA). ONSD: Optic nerve sheath diameter

The mean ONSD (mm) at 0 hour in patients who died was 5.35±0.19 and who survived was 5.2±0.23. This difference was statistically significant (p=0.035) (Figure 2).

A non-significant mild negative correlation was seen between the mean ONSD at 0 hour and APACHE II score, while no correlation was seen between the mean ONSD at 0 hour and SOFA score. Non-significant mild positive correlation was seen between the mean ONSD at 0 hour and duration of ICU stay, whereas no correlation was seen between the mean ONSD (mm) at 0 hour and duration of hospital stay (Table 4).

**Table 4:** Correlation of the mean ONSD at 0 hour with study parameters ONSD: Optic nerve sheath diameter; SOFA: Sequential Organ Failure Assessment; APACHE II: Acute Physiology and Chronic Health Evaluation II; GCS: Glasgow Coma Scale

Correlation of Mean ONSD (mm) at 0 hour	Correlation Coefficient	P-value
SOFA score	-0.027	0.810
APACHE II score	-0.084	0.456
Duration of ICU stay (days)	0.097	0.390
Duration of hospital stay(days)	0.043	0.706

We observed that out of 80 patients, 70 patients had normal ONSD and 10 patients had high ONSD. Twenty-eight-day mortality was higher in patients with high ONSD (30%), as compared to those with normal ONSD (12.86%), but it was not statistically significant (p=0.16). No significant association was found in duration of ICU stay (p=0.77) and duration of hospital stay (p=0.62) with normal and high ONSD (Table 5).

**Table 5:** Association of outcomes with normal and high ONSD Values are expressed as mean ± standard deviation or number (%). *Fisher’s exact test used for categorical variables. ‡Mann–Whitney U test used for continuous variables. ONSD: Optic nerve sheath diameter; ICU: Intensive care unit.

Outcome	Normal ONSD (<5.5) (n=70)	High ONSD (>=5.5) (n=10)	Total	P-value
28-day mortality				
No	61 (87.14%)	7 (70%)	68 (85%)	0.16^*^
Yes	9 (12.86%)	3 (30%)	12 (15%)	
Duration of ICU stay(days)				
Mean ± SD	13.51 ± 5.77	13.9 ± 5.51	13.56 ± 5.71	0.77^‡^
Duration of hospital stay(days)				
Mean ± SD	18.16 ± 6.84	18.7 ± 10.47	18.23 ± 7.3	0.62^‡^

## Discussion

SAE is an acute cerebral dysfunction associated with sepsis and characterized by changes in attention, cognition, behavior, level of consciousness, or neurological function without evidence of direct central nervous system infection. Its pathophysiology is complex and may involve systemic inflammation, endothelial activation, blood-brain barrier disruption, oxidative stress, altered cerebral perfusion, mitochondrial dysfunction, neurotransmitter abnormalities, and neuronal injury. Cerebral edema and increased ICP may occur in selected patients, but they are not universal features of SAE. Ultrasonographic ONSD measurement provides a rapid and non-invasive estimate of possible ICP elevation; nevertheless, it remains an indirect surrogate and should be interpreted within the overall clinical context [14,15].

The present study evaluated serial ONSD measurements, GCS scores, disease-severity scores, and clinical outcomes in 80 patients with SAE. The principal finding was the absence of a statistically significant correlation between ONSD and GCS score at the evaluated time points. Baseline ONSD was also not significantly correlated with APACHE II score, SOFA score, or length-of-stay outcomes. However, mean baseline ONSD was significantly higher among non-survivors than among survivors, although this association was not significant after ONSD was dichotomized using the prespecified 5.5-mm threshold.

The mean age of the patients with SAE in our study was 53.24±13.2 years. In a similar study, Czempik et al. [15] observed that the mean age of the septic shock patients was 65 years, which is higher than in our study. On the other hand, Yang et al. found that the mean age of patients with sepsis was 45 years [13]. In a similar study, Bhide et al. [16] reported that the mean age of nontraumatic neuro-critically ill patients was 64.05±16.8 years. In our study, the majority of patients with SAE were male (52.50%). Similarly, male dominance among patients with sepsis/septic shock has been reported by previous studies [16]. However, one recent study from Poland has reported equal distribution of gender among patients with septic shock [15]. In contrast to these findings, del Saz-Saucedo et al. [17], in a similar study, reported that 86.66% of patients were female. 

The mean age of the study population was 53.24 ± 13.20 years, which was lower than that reported by Czempik et al. and Bhide et al. but higher than that reported by Yang et al. [13,15,16]. These differences may reflect variation in inclusion criteria, geographic populations, infection sources, comorbidity profiles, and disease severity. Men represented 52.5% of the present cohort. Although a similar male predominance has been reported in some sepsis and neurocritical care studies, sex distribution alone is unlikely to explain differences in the ONSD or clinical outcomes among studies.

The GCS is frequently employed for evaluation of neurocritically ill patients, and an association has been reported between poor motor performance with raised ICP and poor prognosis. In patients with difficulty in assessment of the GCS because of ongoing sedatives or paralytic agents, an increased ONSD can be useful as an indicator for increased ICP. We investigated the correlation of the ONSD with the GCS score at various time intervals of 0 hour, 24 hours, 72 hours, fifth day, and on days of deterioration, and found that there was no significant correlation between the ONSD and GCS score at various time intervals (p>0.05). In contrast, Yang et al reported that there was a significant negative correlation between ONSD and GCS score (rs=− 0.666, p<0.001) [13]. Kaur et al. also found that there was statistical significance between poor GCS and increased ONSD in patients with traumatic brain injury [18]. The reason for insignificant correlation in our study can be attributed to the smaller sample size of patients having high ONSD (n=10). 

In our study, the 28-day mortality rate was 15%, which is lower than the mortality rates reported in the studies by Yang et al. [13] (60.7%) and Bhide et al. [16] (43.4%). While determining the association of the mean ONSD at presentation with mortality, we found that the patients who died had a significantly higher mean ONSD; however, when we subcategorized the ONSD levels into normal and high, at a cut-off of ≥5.5, we found that there was no significant association of high ONSD with 28-day mortality. These findings are supported by the study by Yang et al. [13], who mentioned that there is a lack of data indicating that patients with wider ONSD values have increased risk of mortality. Thus, it was suggested that ONSD is not useful to predict the prognosis of SAE patients. 

In our study, the mean duration of ICU stay was 13.56±5.71 days, and the mean duration of hospital stay was 18.23±7.3 days. Similar observations were made in previous studies [13,16]. We found no significant correlation of the mean ONSD with the duration of ICU stay (r=0.097, p=0.390) and duration of hospital stay (r=0.043, p=0.706). Additionally, when we categorized the patients into high ONSD (n=10) and normal ONSD (n=70) based on the cut-off of ≥5.5, there was no significant association of ONSD with ICU stay (p=0.77) or hospital stay (p=0.62). Bhide et al. [16] did not find any significant association between increased ONSD and ICU mortality (P=0.335) in their study, as was observed in our study.

Limitations

This study has several limitations. First, it was conducted at a single center and included a relatively small cohort, limiting external validity. Only 10 patients had an ONSD of 5.5 mm or greater, and only 12 deaths occurred, resulting in limited statistical power and imprecise subgroup estimates. Second, SAE was defined primarily using a GCS threshold, and the manuscript does not describe a standardized diagnostic algorithm incorporating delirium assessment, neuroimaging, electroencephalography, cerebrospinal fluid analysis, or systematic exclusion of metabolic and medication-related causes. Misclassification of SAE is therefore possible. Third, ONSD was not compared with invasive ICP monitoring, lumbar puncture opening pressure, or another reference standard. Consequently, ONSD values cannot be interpreted as direct evidence of intracranial hypertension. Fourth, details regarding operator training, measurement reproducibility, image quality, and intraobserver or interobserver agreement were not available. Fifth, serial measurements may have been affected by missing observations caused by death, discharge, sedation, mechanical ventilation, or clinical improvement, and the number of evaluable patients at each time point was not clearly reported. Sixth, separate cross-sectional correlation analyses did not account for the dependence among repeated measurements obtained from the same patient. Finally, potentially important confounders, including sedative exposure, mechanical ventilation, PaCO₂, serum sodium, renal and hepatic dysfunction, septic shock, vasopressor requirements, and organ-support therapies, were not included in an adjusted analysis. These limitations preclude definitive conclusions regarding the prognostic value of the ONSD in SAE.

## Conclusions

In this prospective cohort of patients with SAE, serial ONSD measurements were not significantly correlated with GCS scores, APACHE II scores, SOFA scores, ICU length of stay, or hospital length of stay. The mean baseline ONSD was higher among non-survivors than among survivors; however, mortality was not significantly associated with the prespecified ONSD threshold of 5.5 mm. This finding should be considered exploratory because of the small number of deaths, the limited number of patients with elevated ONSD, the absence of adjustment for confounding variables, and the lack of a reference standard for ICP. Larger multicenter studies using standardized ONSD acquisition protocols, longitudinal statistical models, and appropriately adjusted outcome analyses are needed to clarify the clinical and prognostic role of the ONSD in SAE.
